# Supplementary material for: Social and clinical risk factors associated with hospitalized COVID-19 patients in Brussels’s deprived and multiethnic areas
Source: PLOS Glob Public Health. 2023 Jul 14;3(7):e0002039. doi: 10.1371/journal.pgph.0002039 (PMC10348590; doi:10.1371/journal.pgph.0002039)
Supplement: S1 Table — (DOCX) [file pgph.0002039.s001.docx]

**S1 Table: Laboratory parameters of the population hospitalized for COVID-19 by nationality groups**

| N (%) | **Belgium n=562** | **EU15**  **n=27** | **EU28**  **(without EU15)**  **n=56** | **Maghreb**  **N=56** | **Sub-Sahara Africa**  **N=47** | **Middle east**  **N=15** | **Other**  **N=19** | **Total**  **N=782** |
| --- | --- | --- | --- | --- | --- | --- | --- | --- |
| **CRP (mg/L)** |  |  |  |  |  |  |  |  |
| <150 | 393 (78.8) | 20 (80) | 41 (78.9) | 42 (79.2) | 31 (72.1) | 13 (92.9) | 14 (73.7) | 554 (78.6) |
| ≥150 | 106 (21.2) | 5 (20) | 11 (21.1) | 11 (20.8) | 12 (27.9) | 1 (7.1) | 5 (26.3) | 151 (21.4) |
| **PaO2 (mmHg)** |  |  |  |  |  |  |  |  |
| ≥60 | 254 (78.9) | 12 (63.2) | 21 (75) | 22 (64.7) | 21 (67.7) | 5 (55.6) | 7 (63.6) | 342 (75.3) |
| <60 | 68 (21.1) | 7 (36.8) | 7 (25) | 12 (35.3) | 10 (32.3) | 4 (44.4) | 4 (36.4) | 112 (24.7) |
| **Lymphocytes** |  |  |  |  |  |  |  |  |
| ≥500 | 133 (28.2) | 25 (100) | 12 (25) | 39 (75) | 38 (86.4) | 10 (71.4) | 13 (68.4) | 270 (40.1) |
| <500 | 339 (71.8) | 0 | 36 (75) | 13 (25) | 6 (13.6) | 4 (28.6) | 6 (31.6) | 404 (59.9) |
| **LDH (IU/L)** |  |  |  |  |  |  |  |  |
| <350 | 129 (55.8) | 9 (37.5) | 12 (54.6) | 23 (54.8) | 15 (38.5) | 8 (66.7) | 2 (15.4) | 198 (51.7) |
| ≥350 | 102 (44.2) | 15 (62.5) | 10 (45.5) | 19 (45.2) | 24 (61.5) | 4(33.3) | 11 (84.6) | 185 (48.3) |
