# Supplementary material for: Social and clinical risk factors associated with hospitalized COVID-19 patients in Brussels’s deprived and multiethnic areas
Source: PLOS Glob Public Health. 2023 Jul 14;3(7):e0002039. doi: 10.1371/journal.pgph.0002039 (PMC10348590; doi:10.1371/journal.pgph.0002039)
Supplement: Attachment — Submitted filename: Response to Reviewers.docx [file pgph.0002039.s002.docx]

Judith Racapé, MSc, PhD

Research center of epidemiology, biostatistics and clinical research

Chair in health and precarity

School of Public Health. CP 598

Université Libre de Bruxelles.

Route de Lennik, 808

1070 Bruxelles – Belgique

Brussels, May 17th 2023

Dear Editor in Chief,

We would like to thank the reviewers for their constructive and relevant comments concerning our manuscript entitled “Social and clinical risk factors associated to hospitalised COVID-19 patients in Brussels’s deprived and multiethnic areas”.We have addressed the concerns and suggestions below.

Yours sincerely,

Judith Racapé

Corresponding Author

**Reviewer #1:**

This topic deals with social determinant of health. However the methodology do not to enable reproducibility, which is a key component in research to enable reproducibility. The magnitude of the problem is not reflecting in the introduction section such as incidence rate of Covid in Belgium, mortality rate from Covid. The research design and study eligibility criteria for participants is missing. The result section is not explained in the detailed as the variables are not spelt out.

Author should include a flow diagram of study participant criteria, The figures are very much needed to improve readability and comprehension of detail. The demographic characteristics should be separated from the hospitalization characteristics.

All result should be interpreted.

We agree that the manuscript can be significantly improved in order to enable reproducibility and we have made several modifications based on the points raised by the reviewer.

- We have added references and a more detailed discussion in the Introduction on the magnitude of the problem in Belgium as suggested also by Reviewer #3 (lines 62-67 and lines 90-99)
- We have provided clarification on the research design and study eligibility criteria for participants in the methodology section, as suggested also by reviewer # 3 (lines 125-128). Additionally, we have included the flow chart of study participants in Figure 1
- We have provided further clarification on data collection (lines 130-134) and described the variables in more detail as suggested also by reviewer # 4. Specifically, we have addressed the age (line 141), nationalities (lines 144-148), region (line 150 and 152-154), and health insurance (lines 159-162) variables.
- We have separated the demographic characteristics from the laboratory parameters. Indeed, due to the substantial number of missing values for LDH and PaO2 in the laboratory parameters, we have chosen not to interpret the results related to these variables. We have made the decision to include the results as a supplementary table. Additionally, we have added clarification in the Methods section to explain this choice (lines 173-176)
- Following the comments of the other reviewer (see below), we have addressed and completed the results section accordingly: lines 205-206, lines 215-216 and lines 219-223.

*References:*

*Luyten J, Schokkaert E. Belgium’s response to the COVID-19 pandemic. Health Econ Policy Law. :1–11*

*Bambra C, Riordan R, Ford J, Matthews F. The COVID-19 pandemic and health inequalities. J Epidemiol Community Health. 2020 Nov;74(11):964–8.*

*Horton R. Offline: COVID-19 is not a pandemic. The Lancet. 2020 Sep 26;396(10255):874.*

*Decoster A, Minten T, Spinnewijn J. The Income Gradient in Mortality during the Covid-19 Crisis: Evidence from Belgium. J Econ Inequal. 2021;19(3):551–70.*

**Reviewer #2:**

The hyothesis is good enough but the scope of the data is limited. The data adds onto global data on Covid-19 but does not propose any new information regarding the compounding and risk factors for Covid-19, that is not available elsewhere for similar populations and could otherwise be extrapolated.

We acknowledge the limited scope of the data, and we have mentioned this limitation in the manuscript. Additionally, we have provided a more detailed discussion on this point in lines 332-338.

One of the significant contributions of our manuscript is the inclusion of all patients, including those without any administrative status such as non-residents, migrants, and refugees. We have highlighted the presence of patients living in shelters without health insurance in our study. This population is often excluded from official statistics and remains understudied. We have clarified this point in the Introduction (lines 100-101) and discussion (line 269, lines 311-314 and lines 333-335) .

This study also points out the challenge in obtaining social and clinical data and emphasizes the importance of routinely collecting both clinical and social data. This is crucial in order to study the significance of social health determinants, particularly among the most vulnerable groups.

Actually data related to migrant/ethnic groups and COVID-19 in Belgium are scarce, with only one existing study focused on excess mortality rates from a population-based perspective. Despite the limited availability of data, this study contributes valuable new information within the context of Belgium As suggested above, we have included directions for future research in the manuscript (lines 332-334).

**Reviewer #3:**

Thank you for submitting your manuscript to PLOS Global Public Health. Overall, I found the manuscript to be well-written, well-organized, and methodologically sound. The study addresses an important and timely topic, and the results interest many readers.

Below are some specific comments and suggestions for improvement:

Introduction:
• The introduction provides an adequate background and rationale for the study. However, it could be strengthened by including a more detailed discussion of the existing literature on COVID-19 and social determinants of health, particularly in the Belgian context**.**

Thank you for this comment.

We have added a more detailed discussion in the Introduction and included some references as suggested also by Reviewer #1 (lines 62-67 and lines 90-99)

Methods:
• The methods section is clear and well-organized. However, including more information about the sampling strategy and how participants were recruited for the study would be helpful. For example, was it a case of enrolling all patients found for the period excluding pregnant women? This example may be the actual case, but not stating it may leave the reader perplexed about what took place.

Thank you for this comment. We have clarified this point in the methodology and added the flow chart as suggested by reviewer # 1 (see above)

Additionally, it would be helpful to know how missing data were handled in the analysis.

In light of the clear limitation posed by the missing data for the laboratory parameters, we have made the decision to present the results in a supplementary table. We have pointed this out in the Methodology (lines 173-176)

Except for laboratory parameter, missing data are less than 3%. However, to handle our missing, we conducted a complete case analysis, restricting the multivariable analysis to individuals with complete information on all variables included in the main analysis. The results obtained from this analysis were found to be similar, indicating that the missing data did not impact the findings.

Results:
• The results section is well-written and presents the findings.
• The authors could also provide more information about the statistical tests used to assess differences between groups and the significance levels of those tests.

Thank you for this comment. We have made the update by adding a column in Table 1 that displays the p-values corresponding to the tests used to assess differences between nationality groups. Indeed, this additional information provides a clearer understanding of the statistical significance of the observed differences. We have added information about the statistical tests used in the methodology: lines 182-184.

Discussion:
• The discussion section is well-written and interprets the results well.
• The authors should also address the limitations of their study more explicitly, although we recognize the limitations presented by word count for certain article types. If possible, they could also suggest directions for future research in this area in greater detail. Once again, word count may limit the ability to address this.

We have addressed more explicitly the limitations and added some directions for future research (lines 332-338).

Conclusion:
• The conclusion is clear and concise.

General comments:
• Overall, the manuscript is well-written and well-organized.

**Reviewer #4:**

Thank you for an interesting deep dive in to the social and clinical risk factors associated with COVID -19 hospitalization in select parts of Brussels. This is needed to drive the discussion on the possible inequities that might drive health and health access in similar pandemics. The manuscript is well written, and clear. It takes a bold and clear look at the possibilities of poorer outcomes for certain nationalities, and highlights deficits in health care outcomes by both clinical and most interestingly, limited social characteristics.
Please find below thoughts and comments that can be addressed to strengthen the already good manuscript.

1. Consider rewriting the title from "Social and clinical risk factors associated to hospitalized COVID-19 patients in Brussels’s deprived and multiethnic areas" to "Social and clinical risk factors associated ***with*** hospitalized COVID-19 patients in Brussels’s deprived and multiethnic areas." "With" is more appropriate grammatically for clarity than "to"

This has been corrected

2. Stylistic and typographical points to aid clarity
   A. Please list the specific objectives rather than placing them in a single long sentence. e.g.
   1. .....
   2. .....

This has been done in the Introduction and the Discussion section.

B. Kindly define EU15, EU28 for the international, uninitiated readership. Is the separation of these 2 EU groups of citizens be justified for this study?

Thank you for pointing this out. We have justified this point in the methodology (lines 144- 148) and added a reference.

*Reference:*

*MYRIA. Nouvelles migrations des pays de l’Est. Focus sur les Roumains, Polonais, Bulgares.; 2016. Belgian Federal Migration Centre; 2016.*

C. Line 146- "tata" should read "data"

This has been corrected.

3. It is very important for the authors to clarify whether they grouped individuals by :ethnicity" or "nationality" which are distinct. Were these nationalities of birth or were, for instance, dark-skinned Belgians of previous African extraction classified as sub-Saharan African in the original dataset? This has huge implications for the study either way.

Thank you. We have used nationality at the hospital admission. We changed “ethnicity” by “nationality” in the manuscript and we have clarified this point in the methodology (line 142) and in the limitation (lines 339-341).

4. In the authors' experience, do patients with temporary health insurance also experience delays in access to tests and health care equivalent to those without health insurance at all? This will be critical to justifying the inclusion of patients with temporary health insurance in the same category of those with no insurance. Kindly justify this in the methods.

Thank you for pointing this out. We have clarified this point in the methodology (lines 159-162) and added a reference

*Reference;*

*Roberfroid D, Dauvrin M, Keygnaert I, Desomer A, Kerstens B, Camberlin C, et al. Quels soins de santé pour les personnes en séjour irrégulier ? Bruxelles: Centre Fédéral d’Expertise des Soins de Santé (KCE); 2015. (Health Services Research (HSR)). Report No.: 257B. D/2015/10.273/111.*

5. **A** brief background of the hospitals and their location would be helpful to readers. Where exactly are they located, how many beds? It would appear (but is unclear from the body of the text) that these hospitals are located in vulnerable settings and are pretty accessible to immigrant communities. What is the profile/diversity of their patients at baseline? Just a brief introduction to these 2 facilities would be helpful.

We have added additional information about the hospitals in the introduction (lines 106-109)

6. Reading the abstract, it is very easy to assume that mortalities are higher for patients from sub-Saharan Africa, even though this is not the case in the body of the manuscript. It is worth mentioning in the abstract around lines 47-48 "Patients from Sub-Saharan Africa were younger, had a higher prevalence of obesity, lacked health insurance, and had the highest proportion of Intensive Care Unit (ICU) admission (27.7%), *but the lowest mortality rates, compared* to other nationality groups." See line 191/192

Thank you, We have modified this point in the abstract

Thank you for this thoughtful manuscript.

**Reviewer #5:**

This paper is well written. However, the authors could consider relating the study to the sustainable development goals (SDG) 3.8, which enjoins member countries to implement and promote the universal health coverage, including financial risk protection, access to quality essential health-care services and access to safe, effective, quality and affordable essential medicines.

Thank you for this comment. We fully agree, and we have incorporated this point into the discussion.

Indeed, this study includes a significant percentage of patients from a foreign nationality without health insurance and we have shown that lack of insurance is a risk factor for ICU admission. Implementing universal health coverage for all individuals, including non-citizens, migrants, and refugees, is a crucial step in reducing barriers to healthcare access for vulnerable populations (lines 311-314)

*Reference:*

*World Health Organization. Regional Office for the Western Pacific. Sustainable development goals (SDGs) : Goal 3. Target 3.8 : Achieve universal health coverage, including financial risk protection, access to quality essential health-care services and access to safe, effective, quality and affordable essential medicines and vaccines for all [poster] [Internet]. WHO Regional Office for the Western Pacific; 2016 [cited 2023 May 14].*
